# Reviews in Medical Education: Advances in Simulation to Address New Challenges in Neurology

**DOI:** 10.1212/NE9.0000000000200042

**Published:** 2023-03-09

**Authors:** Catherine S.W. Albin, J. Palmer Greene, Sara C. LaHue, Prem Kandiah, Arielle M. Kurzweil, Yara Mikhaeil-Demo, Nicholas A. Morris

**Affiliations:** From the Department of Neurology (C.S.W.A., P.K.), Emory University School of Medicine, Atlanta, GA; Department of Neurology (J.P.G., S.C.L.), School of Medicine, and Department of Neurology (S.C.L.), Weill Institute for Neurosciences, University of California, San Francisco; Department of Neurology (A.M.K.), NYU Grossman School of Medicine, New York; Department of Neurology (Y.M.-D.), Northwestern University Feinberg School of Medicine, Chicago, IL; and Program in Trauma (N.A.M.), Department of Neurology, University of Maryland School of Medicine, Baltimore.

## Abstract

Simulation is an engaging modality of medical education that leverages adult learning theory. Since its inception, educators have used simulation to train clinicians in bedside procedures and neurologic emergencies, as well as in communication, teamwork, and leadership skills. Many applications of simulation in neurology are yet to be fully adopted or explored. However, challenges to traditional educational paradigms, such as the shift to competency-based assessments and the need for remote or hybrid platforms, have created an impetus for neurologists to embrace simulation. In this article, we explore how simulation might be adapted to meet these current challenges in neurologic education by reviewing the existing literature in simulation from the field of neurology and beyond. We discuss how simulation can engage neurology trainees who seek interactive, contextualized, on-demand education. We consider how educators can incorporate simulation for competency-based evaluations and procedural training. We foresee a growing role of simulation initiatives that assess bias and promote equity. We also provide tangible solutions that make simulation an educational tool that is within reach for any educator in both high-resource and low-resource settings.

Medical simulation was first pioneered by Barrows, a neurologist, in the 1960s building on rich simulation traditions in the military and aviation industry.^1,2^ Early healthcare simulation focused on diagnosis and clinical reasoning through the use of standardized patients,^1^ but quickly gained prominence in the fields of resuscitation and anesthesiology through the advancement of manikin technology.^3^ Incorporating developments in adult learning theory, including foundational work on experiential learning from Dewey and Kolb,^4^ medical educators have embraced simulation for teaching, assessment, and as a form of translational science to improve patient outcomes.^5^ Notable patient care successes attributable to simulation training include improved operating performance in laparoscopic surgeries,^6^ improved adherence to advanced cardiac life support during in-hospital cardiac arrest events,^7^ and cost-effective reductions in central line–associated bloodstream infections.^8^ Increasing appreciation of the role of cognitive and interpersonal skills (sometimes referred to as nontechnical skills) in patient safety events has expanded the focus of simulation training beyond procedural learning.^9^ There is a growing appreciation for simulation as a method to train clinical teams in the principles of “crisis resource management” (CRM) which is, in brief, the deliberate practice of communication, teamwork, leadership, and situational awareness with an emphasis on streamlining care and preventing errors.^10^

Within neurology, simulation has already been studied as a tool to sharpen clinical performance and improve team performance. It has been implemented to shorten door-to-needle time for ischemic stroke,^11^ promote adherence to status epilepticus algorithms,^12^ improve success rates for lumbar punctures (LPs),^13^ and improve trainees' comfort with EEG interpretation,^14^ while also being leveraged to teach multidisciplinary collaboration,^15^ leadership, professionalism,^16^ and empathetic communication in sensitive topics such as brain death and colleague burnout.^17,18^

We believe that with adaptations, simulation is poised to become an even more powerful teaching strategy for both local and global education, as well as a cornerstone for competency-based assessment. Many *Neurology*® *Education* readers are likely familiar with simulation through participation or even development of simulation scenarios. While we review some of the current and historic applications of simulation in neurology, our focus is also on the innovative ways that simulation might be adapted to the unique challenges and opportunities created by the increasing complexity of healthcare systems, the shift to virtual or hybrid learning, and the transition to competency-based assessment. We also highlight the vast potential for simulation in procedural training, teaching rare pathology, and training future neurologists in the ever-expanding arsenal of neurologic procedures (Table; Figure 1).

**Table:** Education Goals and How Simulation Can be Used to Address Them

Education goal	Challenge for neurologists	Advances in simulation that facilitate addressing these challenge
Competency based evaluations	Establish trainee competency in the era of pass/fail USMLE steps	Development of simulation scenarios that measure clinical acumen and behavior, perhaps with more validity than multiple choice tests Development of simulation curriculum that establishes mastery learning Establishment of validity data in neurologic emergencies More realistic patient simulators
Engaging teaching that requires active learning	Establish curricula that require students to translate knowledge and apply principles	Computer-based simulations Remote simulations that can be accessed anywhere with high-speed Internet Web conference platforms that support hybrid simulation plans that require only a select number of in-person learners
Exposure to rare pathology	Designing more realistic encounters for rare pathology	Advanced patient simulators that incorporate haptics to mimic the “feel” of an examination finding (such as cogwheel rigidity) Augmented reality technology that facilitates displaying rare findings (such as eye movements) on a standard manikin
Onboarding for teleneurology	Develop “webside manner” Attain competency in tele-assessments	Platforms that enable remote simulation Virtual simulation scenarios and evidence that supports their use and trainee improvement
Team training using principles of crisis resource management	Onboarding and training of the many providers who care for patients with neurologic emergencies	High-fidelity and immersive simulation environments Computer-based simulations Platforms that enable remote simulation
Evaluation of bias	Facilitate the recognition of bias Provide culturally sensitive care	More racially and ethnically diverse simulation manikins New simulation body adaptions and engineered masks that allow representation of community diversity
Procedural training	Establish trainee competency without harm to patients	Low-cost 3D-printed lumbar puncture models Augmented reality–enhanced lumbar puncture and mechanical thrombectomy trainers Simulators that teach learners evaluate other neuropathology (optic nerve sheath diameter and transcranial doppler trainers)
Globalization of neurology training	Establish Neurology curricula and educational initiatives in lower-resource areas while still ensuring high performance	Lower-cost manikins and low-cost 3D-printed simulators for task training models Virtual conferencing platforms Virtual simulation scenarios

Abbreviations: 3D = 3-dimensional; USMLE = United States Medical Licensing Examination.

**Figure 1:**
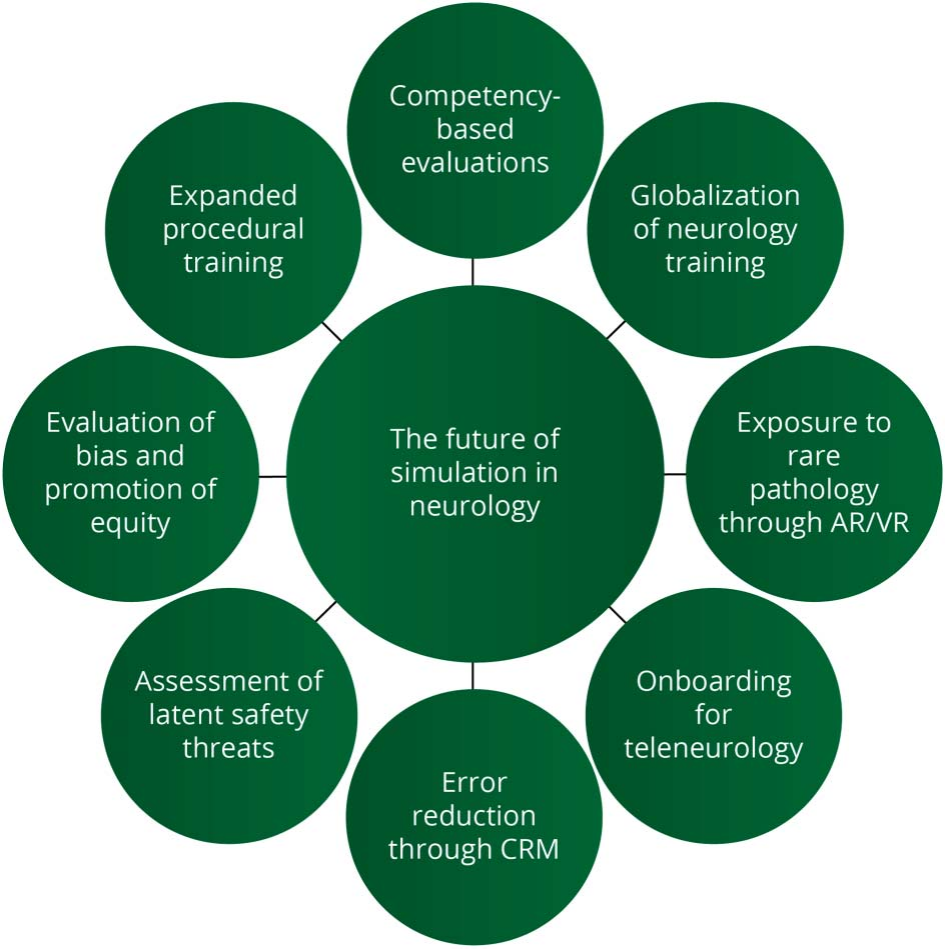
The Future of Simulation in Neurology An overview of the many areas in neurology training where simulation may be applied. AR = augmented reality; CRM = crisis resource management; VR = virtual reality.

## Simulation for Neurologic Emergencies and Principles of CRM

Systems of clinical care are becoming increasingly complex. Nowhere has this been more evident than in the care of neurologic emergencies. At every juncture of care, the teams who triage and manage neurologic emergencies must act expediently. Essentially, these teams must excel at the fundamental principles of CRM.

CRM is the framework for the nontechnical skills necessary to manage a crisis safely and effectively. Principles of CRM include situational awareness, triage and prioritization, cognitive load reduction, role clarity, communication, and debriefing.^10^ The framework was piloted by the aviation industry, initially under the moniker “crew resource management,” but has been adapted to medical emergencies, particularly within the field of anesthesia and emergency medicine.^19,20^ Recently, the application of CRM principles has been suggested as a way to expedite care and reduce medical errors during stroke codes.^21^

Given the rich history of using simulation for CRM and team training, neurology educators have already demonstrated successes in the application of these principles to neurologic emergencies. For example, the Simulation-based Training of Rapid Evaluation and Management of Acute Stroke (STREAM) intervention in Germany recognized that a well-organized stroke team was crucial to swift administration of thrombolysis.^11^ The researchers' introduced principles of CRM and simulation during 2 full-day trainings at multiple sites. After this intervention, door-to-needle time improved by 5 minutes in the CRM-trained teams compared with that in controls. Similar gains were reported by a Norwegian stroke center.^22^ However, a study using CRM simulation training to improve care of patients in the Epilepsy Monitoring Unit did not find any benefit among physician-nursing dyads perhaps because of skill decay between training and assessment.^23^ For low frequency, high acuity of events such as status epilepticus, some authors suggest embracing a “seizure code” model and use CRM training for these teams to reduce inaccurate detection and inappropriate medication dosing.^24^

Debriefing is a crucial component of CRM and can be leveraged to expose latent safety threats. For example, a New York City multihospital system used an in situ “code stroke” to capture several latent safety events that were discovered during the debrief. Discoveries included how difficult it was to obtain an accurate patient weight as well as provider confusion regarding how best to re-evaluate a patient after treatment. These difficulties were brought to hospital leadership for rectification before activation of the site collaboration.^15^

Principles of CRM and deliberate practice through simulation have other potential applications to be explored in neurology. To date, few studies include any family members in simulation training, but future work could incorporate these caregivers into training for neurologic emergencies that occur as exacerbations of chronic disease—such as the care of patient with poorly controlled epilepsy or myasthenic gravis. Alternatively, focusing on process and equipment evaluation, CRM principles could also help in onboarding providers to new models of care such as mobile stroke units, ideally prior to launching these services. Finally, simulation and CRM principles have been less well studied in other neurologic emergencies that require teams to rapidly recognize, triage, and treat the patient to prevent neurologic injury. For example, although simulations have been used to train providers to manage neuromuscular emergencies^25^ and cerebral herniation,^26^ these simulations have focused only on 1 provider, not interdisciplinary teams.

## Simulation and Remote Learning

Although many emergency teams continued to work and train in person during coronavirus disease 2019 (COVID-19) surges, the pandemic forced many medical training programs across the globe to adapt education curricula to remote models. Simulation training activities were also disrupted, forcing educators to experiment with different strategies for engaging learners and equipping them with the skills that had previously been taught through simulation. Although most restrictions to in-person learning have lifted at this time, time constraints remain, and hybrid or virtual training may still be preferable in some cases. Creative solutions to increase simulation's accessibility remain relevant. Examples of simulation adaptations include the development of online, computer-based simulations, the engineering of “telesimulations” or “remote simulations,” and the creation of hybrid (in person plus distanced) simulations.

**Figure 2:**
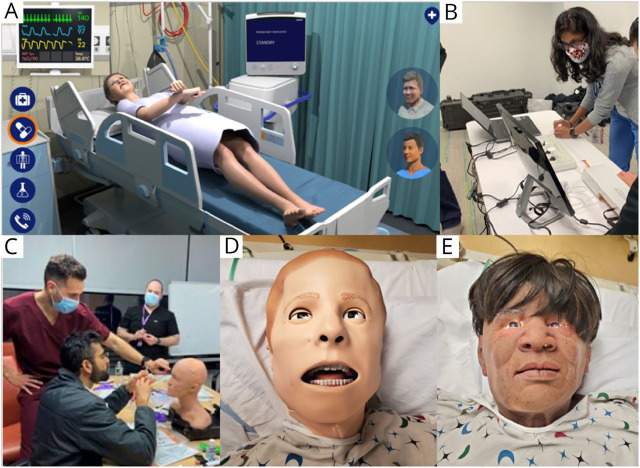
Advances in Simulation Technology and Applications (A) The Neurocritical Care Society's Emergency Neurologic Life Support now offers computer-based simulation of 6 neurologic emergencies, including refractory status epilepticus. (B) A neurointerventionalist oversees a resident practicing catheter manipulation using a thrombectomy simulator. (C) A resident practices botulinum toxin injection for migraine relief on a training manikin. (D, E) Masks can transform existing manikins to better represent diverse patient populations and promote health equity and inclusion.

An example of a fully online simulation comes from the Neurocritical Care Society, which recently developed computer-based simulation cases as an interactive adjunct to its Emergency Neurologic Life Support course. These 6 simulation modules extricate interactive learning of neurologic emergencies from high-resourced simulation centers, making them available to any global learner with computer access (Figure 2A). Virtualized team simulations have also been tested by pediatric emergency medicine providers using a free, open-access platform, the “American College of Emergency Physicians SimBox” (emergencysimbox.com). Some of these scenarios include caring for neurologic emergencies such as seizures or altered mental status.^27^ In addition to providing seizure simulations, the site also curates open-access webinars, podcasts, and decision algorithms, thus promoting asynchronous learning. Following implementation in 36 states, participants reported an increased readiness for pediatric emergencies.^28^

Online, screen-based simulation cases have also been developed to help teach novice EEG readers to recognize commonly encountered findings. Critical care provider participants without a background in electrophysiology felt that simulated cases aided EEG interpretation and treatment decisions.^14^ We envision similar screen-based cases for EMG/nerve conduction study (NCS) practice, woven into existing self-paced learning curricula.^29^

Beyond screen-based initiatives, the model in which the learner cares for a simulated patient or completes a simulated training in an environment remote from the teacher has been termed “telesimulation” or “remote simulation.” During the COVID-19 pandemic, telesimulation was first used to teach proper PPE and critical care techniques; it was found to be both feasible and well received.^30^ In 1 study, learners logged onto a virtual platform, practiced using personal protection equipment, and demonstrated how to assist in airway management; the recording was submitted to a trainer for feedback. For emergencies that required team training, Emergency Medicine clinicians explored drastically reducing the number of in-person learners and engaging remote learners in the simulation by having these residents critique the live participants and debrief after the assessment.^31^ Contrary to what was expected, all the learners who participated in the remote “critique and debrief” believed that the virtual experience was better or the same as an in-person simulation. This study did not measure any learning outcomes but at least suggests students' openness to engaging in remote simulation.

Given the success in critical care and emergency medicine, some neurologists have also embraced this paradigm. One institution piloted remote objective structured clinical examinations (OSCEs) to allow students to practice delivering sensitive information to patients.^32^ In this exercise, students disclosed a diagnosis of nonepileptic events to a standardized professional “patient” via a teleconferencing platform. The remote simulation was perceived positively; every learner believed that the simulation was useful and should be repeated in future years.

Projects exploring remote and online simulation have noted that the ease of scheduling this method allows for more frequent observations. Many remote or fully virtual paradigms also bypass the steepest costs associated with an in-person simulation training. However, there are drawbacks to using remote simulation. Fully online simulations require high-speed Internet access, and the degree of hands-on participation is limited. For remote observation simulation and telesimulation, the technological requirements are significant: high-quality webcams, available web-conferencing platforms, high-speed Internet to support their use, screen-sharing software, and high-quality microphones.^33^ These technical requirements may be less for remote OSCEs and higher for interprofessional education and manikin-based simulations when multiple participants are working as a team or when multiple angles are needed to fully assess the participant(s). Another potential limitation of this design is that these situations may still provoke anxiety among learners without the same degree of support as in-person simulation. It is critical that as more remote simulations are piloted in neurology, educators explore learners' acquisition of knowledge as well as learners' attitudes toward this novel platform for simulation.

## Simulation and Teleneurology

The COVID-19 pandemic not only introduced the need to educate differently but also changed the way many neurologists deliver care. There has been a dramatic increase in teleneurology, and as such, it is imperative that residencies incorporate teleneurology education into their curricula. The American Academy of Neurology Telemedicine Work Group provides a list of competencies, milestones, and resources and recommends simulation training.^34^ Despite this, before the COVID-19 pandemic, training programs had scarcely adopted telemedicine training for neurology trainees. Only 1 program had developed a formal curriculum for residents in which they participated in didactics and rotated in a telemedicine clinic.^35^ Simulation in teleneurology offers an opportunity to hone skills in “webside” manner and practice the teleneurology examination.^36^ Much of what is known about simulation in this context is due to vascular neurology's early adaptation of simulation training for telestroke. One telestroke education program used simulation training and real-time clinical practice to prepare their residents to conduct prehospital assessments of patients with potential stroke.^37^ This preassessment resulted in shortened time to treatment once patients arrived at the treatment center. In a different program, education researchers discovered that without any simulated practice of telestroke, vascular neurology fellows would initially take 9 minutes longer from receiving the stroke page to administering thrombolytics than attendings. Over time, the fellows' speed did improve—every 14 consults improved the door-to-needle time by 1 minute.^38^ Knowing that repetition and familiarity could improve the page-to-needle time, some vascular neurology programs have already piloted Simulation Learning, Education and Research Network curricula, which allow their providers to practice telestroke consultations with a standardized patient before triaging live cases.^36^ The study to validate the effect of this intervention is ongoing.

## Simulation for Training and Development in Global Neurology

The potential benefit of simulation goes beyond regional care team networks. Global disparities in neurology are significant. In the last decade, an increasing number of neurology residency programs have facilitated global neurology experiences for trainees; however, neurologic care in resource-limited settings remains challenging partly due to the sporadic nature of neurologist visits.

Studies about the role of simulation to disseminate neurologic knowledge are sparse. However, 1 proposed solution has been the use of blended learning curricula in countries where neurology or subspecialty neurology training is lacking. In a model developed in Pakistan, movement disorder training was directed through an e-learning curricula with supervision and project facilitation remotely by a US board-certified movement disorders neurologists and local physicians.^39^ In this model, the fellows were evaluated by a knowledge assessment, but simulation could potentially offer a more valid assessment of the fellows' performances.

In a non-neurology context, global programs that focus on skill training with low-fidelity manikins have also been highly effective and have had measurable impact. The Neonatal Resuscitation Program has arguably set the standard for a globally transferrable simulation program aimed at reducing infant and maternal mortality. Studies have shown that this training improves provider knowledge and skill but, more importantly, has correlated with the decrease in asphyxia-specific mortality.^40^ Much of the success of the program has been due to the strong collaboration between professional associations and the ministry of health in the target nation. Furthermore, these initiatives often begin with comprehensive training throughout the public health system, with a trickle-down effect to other healthcare providers. This success highlights the impact of partnering with and having buy-in from local health authorities.^41^ This program provides a blueprint for how neurologic simulations that are aimed at improving stroke or status epilepticus management might be adapted to the global stage. However, there is a danger to a plug-and-play approach for using existing simulations. Educators must be conscious of resource limitations, the cultural context and norms of treatment, and the differences in health systems.^42^

## Simulation in Competency-Based Assessment

Whether locally or globally, ensuring trainees are equipped to manage neurologic emergencies and complicated cases is a cornerstone of training. In 2015, the American Board of Psychiatry and Neurology formed a joint initiative with the Accreditation Council for Graduate Medical Education (ACGME) to develop Milestones that provided a framework for the assessment of neurology residents. This was recently updated in July 2021 (Milestones 2.0).^43^ Despite guidance on *what* needs to be assessed at each level, the Milestones initially did not provide guidance on *how* milestones should be assessed. Residency programs thus often relied on the end-of-rotation evaluations and faculty feedback, which may be flawed due to subjective faculty opinions^44^ and because they are predicated on chance clinical encounters. These may be particularly inaccurate in assessing resident's performance in neurologic emergencies or in other situations when oversight is provided remotely.^45^

Simulated clinical scenarios have already been developed to evaluate trainee performance in some critical, high-stake emergencies and provide evidence for areas of improvement. For example, in a simulated acute stroke requiring tissue plasminogen activator administration, followed by hemorrhagic conversion, trainees made frequent errors in managing hemorrhagic transformation—a low-frequency, high-acuity event.^46^ Similarly, in a study of graduating neurology residents from 3 academic centers, trainees performed poorly in identifying and managing status epilepticus using a simulation-based assessment.^47^ Their score in the simulation was then compared with their assigned Milestones at graduation. Despite poor performance in the simulation, all residents had received a “Ready to Graduate” as their Milestone level. There were no significant associations between simulation performance and the level of Milestone assigned at graduation. This suggests that end-of-rotation evaluations alone are inadequate for assigning Milestones for low-frequency, high-acuity events.

Furthermore, management of complicated neurologic patients requires more than medical knowledge. Trainees must also demonstrate communication skills, quick thinking, mental flexibility, and the ability to perform under stress. Assessment of these subcompetencies can be difficult in the clinical environment, but simulation offers an authentic alternative for observation. Thus, simulations to assess graduation readiness can be used to evaluate both technical and nontechnical skills that would eliminate the reliance on chance clinical scenarios and evaluator bias and subjectivity. Given all these benefits, the Milestones 2.0 supplement material now lists simulation as a tool for competency assessment.^43^

If simulation is to be used for competency assessment, then establishing standards for minimum competence and validating simulation to measure competency will be important. Work to validate simulation is ongoing; for example, a recent study published validity evidence for a 10-case simulation-based assessment addressing core neurocritical care emergencies.^48^ This study used the Messick framework of validity evidence to develop evaluative simulations for neurologic emergencies. Similarly, the study of residents' competency in the management of status epilepticus emphasized the importance of developing expert consensus for expected behavior using the Angoff method and Mastery Angoff method.^49^ These methods allowed the authors to determine a minimum passing score, a process that improved reproducibility and the validity of findings.

Given the endorsement by the ACGME, simulation for summative evaluations may soon be the most widely adopted form of simulation. It will be imperative for educators in neurology to study the impact of these evaluations—describing the scenarios that are trialed, how validity is established, and how residents are remediated if they do not pass. These experiences should continue to inform the teaching community on strengths and challenges to simulated encounters. The future of simulation will depend not just on the individual residents' performance but from our community's understanding of what has been trialed, how interventions translate into clinical changes, and how obstacles are overcome.

## Emerging Technology for High-Fidelity Simulation of Neurologic Pathology

A common challenge in neurology training is correctly identifying a physical examination finding that one has not encountered before in practice. Some neurologic pathologies, such as palatal tremor and periodic alternating nystagmus, are uncommon enough that they may be seen only a handful of times in the course of residency training or not at all.^49,50^ Innovations in simulation, such as next-generation manikins and augmented reality/virtual reality (AR/VR) simulation may allow authentic mimicry of less common examination findings and neurologic syndromes, allowing trainees to hone their diagnostic acumen in a controlled setting.

Previously, clinical neuroscience instructors using simulation teaching have had to work around manikins' inability to imitate a focal neurologic deficit. However, next-generation manikins such as the HAL S5301 multidisciplinary patient simulator (Gaumard Scientific) are able to simulate motor findings such as facial droop, extremity weakness, and the reflex examination to recreate realistic presentations of stroke and traumatic brain injury.^51^ Advances in computer language enable manikins to engage in conversational speech with examiners and respond to their history-taking questions. We envision future manikins with advanced robotics providing haptic and programmed neurologic findings, so that residents may experience cogwheel rigidity or “give-way” strength.

Similarly, AR and VR innovations are increasingly being applied to neurology training. Two related concepts, AR refers to using smartphone or wearable visualization hardware such as Google Glass (Google LLC, Mountain View, CA) to apply a digital overlay to one's view of the real world, while VR refers to a fully simulated, dynamic 3-dimensional (3D) environment that the user can interact with in a way that seems real. AR and VR have both been shown to enhance learning in some areas of neurology, including preclinical neuroanatomy in small, single-center studies.^52^ Future applications could include more immersive simulation or the overlay of examination findings to a blank manikin. Such would allow residents to practice rare maneuvers such as the Head Impulse-Nystagmus-Test of Skew examination with real-time “normal” and “abnormal” findings.

Outside of neurology, AR has been used to overlay a presentation of respiratory distress on a manikin with high satisfaction among learners.^53^ However, research thus far investigating the effects of AR/VR implementations is limited by a dearth of robust studies with rigorous methodologies. One systematic review and meta-analysis of computerized virtual patients in healthcare provider education showed improved knowledge outcomes and clinical reasoning compared with no intervention.^54^ Interpretation was limited by large variability in effect sizes, lack of randomization, and paucity of key methodologic details in manuscripts. To address the lack of consensus quality measures for assessing the effectiveness of AR interventions, 1 group proposed an analytical model that takes into account (1) resemblance to reality, (2) content relevance, (3) validity of skill measurement, (4) concordance with “gold standard” training method scoring, and (5) correlation with actual performance.^55^ Notably, of the 36 studies of AR applications in medical education identified in their systematic review, none of them provided sufficient validity assessments to evaluate them on all 5 criteria. Thus, although advances in technology have the potential to dramatically improve on traditional limitations of manikin-based simulation, much work is needed to study the application in neurology and validate the application of this technology.

## Simulation and the Future of Procedural Training in Neurology

Procedural training comprises a large portion of simulation training and research. In landmark studies of central venous catheter insertion, multiple studies demonstrated cost-effective reductions in central line–associated bloodstream infections in real patients through simulation training.^56^ In neurology, the use of LP trainers has improved the first LP success rate of medical students who receive training compared with controls.^57^ Historically, LP task trainers cost thousands of dollars, a prohibitive expense to less-resourced educators, and few were ultrasound compatible. Now, 3D printing technology permits fabrication of ultrasound-capable LP simulators that can be modified to represent specific pathologies or body habitus for as little as $25–30 per simulator.^58^

At the opposite end of the cost spectrum, AR-enhanced LP trainers allow practice on different anatomies, provide more realistic haptic feedback, encourage appreciation of complex 3D anatomy, provide audiovisual feedback relaying patient emotions, and track performance metrics (InSimo, Strasbourg, France). Realistic simulators that mimic fluoroscopy-guided LPs have also been developed and may assist in training budding interventional neuroradiologists.^59^

Besides LP task trainers, clinical neurology training has not generally embraced simulation for procedural training. However, neurology-facing specialties have created do-it-yourself (DIY) simulators that may be adaptable to neurology trainees and educators. For example, fundoscopy simulators, first developed by ophthalmology educators, were recently described as important adjuncts to fundoscopy training in neurology residents.^e1^ Emergency medicine educators have developed a simulator for ultrasound-based evaluation of optic nerve sheath diameter, which could be especially valuable in low-resource areas where traditional neuroimaging technologies may not be present.^e2^ A recently engineered transcranial Doppler simulator (Sheehan Medical LLC, Mercer County, WA) may similarly allow learners to acquire skill in detecting large vessel occlusions with point-of-care technology, useful in low-resource settings where CT angiography is not available. Finally, interventional neuroradiologists have used advanced simulators to hasten the learning curve in performing thrombectomy, an advancement that may facilitate neurology residents' entry into neurointerventional fellowships (Figure 2B).^e3^

More opportunities exist to engage trainees in simulation-based learning in outpatient-focused subspecialities. A study of procedures among neurologists from 2000 to 2010 found that the fastest growing procedural volume was in headache medicine.^e4^ Unfortunately, most trainees stick to patients without first practicing on a simulator.^e5^ Inexpensive and reproducible models that realistically model tissues, nerves, and fascial planes under ultrasound, previously used to teach ultrasound-guided regional anesthesia, are now being repurposed to teach botulinum toxin injections or cranial nerve blocks (Figure 2C).^e6^ Botulinum toxin has also become the primary treatment of focal or segmental dystonia. Trainees likely would benefit from simulation-based injection training, especially in sensitive areas such as the pretarsal orbicularis oculi for blepharospasm. Finally, EMG/NCS simulators could be easily developed to accelerate procedural learning and attenuate patient discomfort. Such simulators would allow for self-directed, on-demand procedural practice, consistent with best practice in adult learning theory.

## Simulation in the Assessment of Bias and a Component of DEI Training

The educational objectives in medical simulation curricula prioritize clinical (e.g., diagnosis and management of acute stroke) or technical (e.g., intubation) skills, while ignoring the responsibility to recognize biases that may be entwined with the curricula itself. These biases may be self-reinforcing, harmful to both trainees and patients, and may serve to counteract diversity, equity, and inclusion (DEI) ideals. Simulation curricula may be thoughtfully restructured to promote DEI initiatives, to facilitate assessing one's own biases, and to provide culturally competent care that advances everyday interpersonal interactions.

Simulation curricula may contain implicit biases from its creators, educational norms, the medical establishment, and the broader culture within which all these operate. This can result in cognitive biases for trainees that may affect performance during real clinical scenarios.^e7^ For example, certain patient populations may be excluded from simulation scenarios, such as pregnant individuals, though they may require specialized management by nonobstetricians during medical emergencies.^e8^ Likewise, simulation models have traditionally lacked diversity in skin tone, age, and body type, favoring White, young, muscular physiques.^e9^ Select companies now engineer masks, skins, and body adaptations to modify preexisting homogenous mannequins into diverse simulated patients who better represent the patient community (Figure 2, D and E).

An underused benefit of simulation curriculum is the promotion of DEI initiatives for medical staff. Training on providing culturally sensitive care benefits future encounters with patients who often experience healthcare inequities. For example, healthcare providers can role-play clinical encounters with sexual and gender minority individuals, including using preferred pronouns, to prevent heteronormative microaggressions.^e10^ Simulation can also provide medical trainees with tools to navigate bias and discrimination directed toward them by patients or colleagues in the workplace.^e11^ Previously developed anti-bias communication curricula were demonstrated to be feasible and beneficial for surgical residents.^e12^ Simulation provides a means for privately self-assessing personal bias as well. In 1 study, evaluations of medical staff perceptions of a video-recorded lead surgeon in different operating room scenarios revealed biases in sex, race, and age.^e13^ Insight into these biases can then inform participation in subsequent interprofessional workshops and provide personal benchmarks for growth.

Educators should consider ways that simulation curricula can advance DEI principles to benefit patients, trainees, and staff. The creators of simulation curricula have a responsibility to be cognizant of biases that may be promoted in their tools. Efficacy in training can be subsequently assessed using either later simulations or by surveying participants about their experiences during on-the-job encounters presimulation and postsimulation.

## Leveraging Advances to Overcome Traditional Barriers

Despite the many ways simulation can address challenges in training and assessment, educators in neurology have been reluctant to embrace simulation.^e14^ Critics of simulation call attention to the challenges in creating high-fidelity neurologic simulations, learner anxiety, the cost associated with simulation programs, as well as the need for expertise in planning and debriefing. Fortunately, many of these barriers are being addressed.

Manikins have obvious limitations when it comes to the physical exam. However, advances in VR and AR are poised to recreate neurologic exam findings more realistically and more affordably. But not all simulations require high-fidelity technology. In a pilot study comparing 2 educational simulation platforms for the diagnosis and management of neurologic emergencies—one with a manikin and the other with a standardized patient—residents in both groups grained equivalent confidence and knowledge despite lower physical examination fidelity in the manikin platform.^e15^ Results may differ when the learning objective is development of physical examination skills or localization because the primary objective of the pilot study was implementation of management protocols for neurologic emergencies. Understanding when and how fidelity affects knowledge acquisition will allow proper resource allocation to high-fidelity models when there is an expected benefit.

Learner anxiety and the perception that simulation is an attempt to deliberately expose trainee weakness has been described as a limitation and drawback of simulation. Wijdicks et al. describe the flawed comparison of simulation and “pimping”—noting that simulation should instead be viewed as complementary to the Socratic method to help learners draw parallels and connects between didactic and clinical experiences.^e16^ Nevertheless, learners do frequently describe feeling nervous and stressed by simulation scenarios. This may be more pronounced if the simulated patient dies, which is a controversial event in the simulation literature and should be avoided with more novice learners.^e17^ It is imperative that simulations occur in a culture of safety in which feedback is not punitive. Establishing a safe container or a “basic assumption”^e18^ that all learners are doing their best and participating in the spirit to learn is crucial in developing a positive experience for learners.

Cost and access to specialized simulation centers are other barriers to adoption of simulation, but here too, technologic advances are expanding access. 3D-printed models can dramatically lower the cost of a task trainer for LP,^58^ while partnering with equipment vendors may offer trainees the ability to practice EMG/NCS, neurotoxin injections, and other procedures with little cost. For nonprocedure training, computer-based simulations and “remote simulations” via a video conferencing platform may offer more accessible price point by bypassing the cost of a simulation center. When in-person events are deemed preferable, new iOS and Android applications make replicating a simulation center feasible at low cost. Applications such as *Simpl* allow vital signs to be displayed on a tablet or smartphone and remote controlled with a linked smartphone. These applications can eliminate the need for access to a simulation center, and in non-neurologic contexts, use of these applications has resulted in similar student outcomes.^e19^

Creation and implementation of a simulation curriculum requires expertise in case creation and production, as well as familiarity with debriefing techniques that neurology departments may lack. New medical education–focused platforms, such as *MedEd Portal*, publish neurology-focused simulation cases. These published, peer-reviewed cases include resources and checklists that can be adapted to meet educators' objectives. As more neurology educators embrace simulation, development of a repository of curated, vetted cases will facilitate simulation-based education and could allow educators to share grading rubrics. Such collaboration will be needed to adequately power studies that measure clinically important outcomes after simulation training.

Finally, although simulation courses do require a higher faculty to student ratio, faculty facilitators need not be content experts; learner-centered approaches help students actively develop their own knowledge and skills in lieu of passively receiving expert didactic lectures.^e20^ Senior residents or fellows may substitute for faculty as near-peer teachers in many roles, such as implanted participant or checklist assessor. Simulation technologists with debriefing expertise can lead a discussion and facilitate reflection even without a deep knowledge of the content. In fact, they may do so better than faculty content experts without skill in debriefing.^e21^ There are multiple free resources for educators who want to improve their debriefing skills including resources from the Center for Medical Simulation and Debrief2learn.org. With technological advances and online resources, even programs with smaller budgets, no access to simulation facilities, and limited debriefing experience can plan and implement an engaging experience for formative training or a realistic environment for summative evaluation.

## Conclusion

Simulation is poised to transform neurology training, assessment, and care delivery. Leveraging the principles of CRM, team training simulations can be used to streamline processes and reduce cognitive errors in neurologic emergencies. Advances in “remote simulation” and hybrid curriculums will enable training in groups with limited ability to meet in person. Furthermore, these virtual and hybrid models may promote the use of healthcare simulation beyond highly resourced academic centers. Simulation also holds great promise in the development of competency-based assessment and should continue to be validated as a grading model. At the leading edge, advanced robotics and VR or AR will expose trainees to life-like examination findings and neurologic syndromes that they rarely encounter in brief clinical rotations, but ought to experience firsthand. Lower cost DIY models will improve procedural skill by facilitating sustained, deliberate practice, even in remote, low-resourced settings. Making just as much of an impact, simulation will address critical DEI deficiencies to expose biases and provide culturally competent care. Although not without some limitations, by building on pioneering studies, simulation offers tomorrow's educators a dynamic range of formats to teach and assess both virtually and at the bedside.

## Appendix Authors

**Table:**

Name	Location	Contribution
Catherine S.W. Albin, MD	Department of Neurology, Emory University School of Medicine, Atlanta, GA	Drafting/revision of the article for content, including medical writing for content; study concept or design
J. Palmer Greene, MD	Department of Neurology, School of Medicine, University of California, San Francisco	Drafting/revision of the article for content, including medical writing for content; study concept or design
Sara C. LaHue, MD	Department of Neurology, School of Medicine, and Department of Neurology, Weill Institute for Neurosciences, University of California, San Francisco	Drafting/revision of the article for content, including medical writing for content; study concept or design
Prem Kandiah, MD	Department of Neurology, Emory University School of Medicine, Atlanta, GA	Drafting/revision of the article for content, including medical writing for content; study concept or design
Arielle M. Kurzweil, MD	Department of Neurology, NYU Grossman School of Medicine, New York, NY	Drafting/revision of the article for content, including medical writing for content; study concept or design
Yara Mikhaeil-Demo, MD	Department of Neurology, Northwestern University Feinberg School of Medicine, Chicago, IL	Drafting/revision of the article for content, including medical writing for content; study concept or design
Nicholas A. Morris, MD	Department of Neurology, Program in Trauma, University of Maryland School of Medicine, Baltimore	Drafting/revision of the article for content, including medical writing for content; study concept or design

## Glossary

3D: 3-dimensional
ACGME: Accreditation Council for Graduate Medical Education
AR: augmented reality
COVID-19: coronavirus disease 2019
CRM: crisis resource management
DEI: diversity, equity, and inclusion
LP: lumbar puncture
NCS: nerve conduction study
OSCE: objective structured clinical examination
VR: virtual reality

## References

[1] Barrows HS. An overview of the uses of standardized patients for teaching and evaluating clinical skills. AAMC. Acad Med. 1993;68(6):443-451; discussion 451-443. doi:10.1097/00001888-199306000-00002. 8507309

[2] Rosen KR. The history of medical simulation. J Crit Care. 2008;23(2):157-166. doi:10.1016/j.jcrc.2007.12.004. 18538206

[3] Issenberg SB, McGaghie WC, Hart IR, et al. Simulation technology for health care professional skills training and assessment. JAMA. 1999;282(9):861-866. doi:10.1001/jama.282.9.861. 10478693

[4] Kolb DA. Experiential Learning: Experience as the Source of Learning and Development. FT Press; 2014.

[5] McGaghie WC, Draycott TJ, Dunn WF, Lopez CM, Stefanidis D. Evaluating the impact of simulation on translational patient outcomes. Simul Healthc. 2011;6(suppl)(7):S42-S47. doi:10.1097/sih.0b013e318222fde9. 21705966 PMC3153601

[6] Sroka G, Feldman LS, Vassiliou MC, Kaneva PA, Fayez R, Fried GM. Fundamentals of laparoscopic surgery simulator training to proficiency improves laparoscopic performance in the operating room-a randomized controlled trial. Am J Surg. 2010;199(1):115-120. doi:10.1016/j.amjsurg.2009.07.035. 20103076

[7] Wayne DB, Didwania A, Feinglass J, Fudala MJ, Barsuk JH, McGaghie WC. Simulation-based education improves quality of care during cardiac arrest team responses at an academic teaching hospital: a case-control study. Chest. 2008;133(1):56-61. doi:10.1378/chest.07-0131. 17573509

[8] Cohen ER, Feinglass J, Barsuk JH, et al. Cost savings from reduced catheter-related bloodstream infection after simulation-based education for residents in a medical intensive care unit. Simul Healthc. 2010;5(2):98-102. doi:10.1097/sih.0b013e3181bc8304. 20389233

[9] Gawande AA, Zinner MJ, Studdert DM, Brennan TA. Analysis of errors reported by surgeons at three teaching hospitals. Surgery. 2003;133(6):614-621. doi:10.1067/msy.2003.169. 12796727

[10] Duggan LV. Optimizing crisis resource management to improve patient safety and team performance: a handbook for acute care health professionals. Can J Anesth. 2018;65;139-140. doi:10.1007/s12630-017-0958-1.

[11] Bohmann FO, Gruber K, Kurka N, et al. Simulation-based training improves process times in acute stroke care (STREAM). Eur J Neurol. 2022;29(1):138-148. doi:10.1111/ene.15093. 34478596

[12] Sutter R, Tisljar K, Opić P, et al. Emergency management of status epilepticus in a high-fidelity simulation: a prospective study. Neurology. 2019;93(19):838-848. doi:10.1212/wnl.0000000000008461. 31594860

[13] Barsuk JH, Cohen ER, Caprio T, McGaghie WC, Simuni T, Wayne DB. Simulation-based education with mastery learning improves residents' lumbar puncture skills. Neurology. 2012;79(2):132-137. doi:10.1212/wnl.0b013e31825dd39d. 22675080 PMC3390539

[14] Fahy BG, Vasilopoulos T, Chau DF. Use of flipped classroom and screen-based simulation for interdisciplinary critical care fellow teaching of electroencephalogram interpretation. Neurocrit Care. 2020;33(1):298-302. doi:10.1007/s12028-020-00985-5. 32424536

[15] Bentley S, Feldman N, Boehm L, et al. Stroke code from EMS to thrombectomy: an interdisciplinary in situ simulation for prompt management of acute ischemic stroke. MedEdPORTAL. 2021;17:11177. doi:10.15766/mep_2374-8265.11177. 34504950 PMC8380761

[16] Kurzweil AM, Lewis A, Pleninger P, et al. Education research: teaching and assessing communication and professionalism in neurology residency with simulation. Neurology. 2020;94(5):229-232. doi:10.1212/wnl.0000000000008895. 31959708

[17] Morris NA, Zimmerman EE, Pozner CN, Henderson GV, Milligan TA. Brain death determination: an interprofessional simulation to determine brain death and communicate with families focused on neurology residents. MedEdPORTAL. 2020;16:10978. doi:10.15766/mep_2374-8265.10978. 33005731 PMC7521065

[18] Stainman RS, Lewis A, Nelson A, Zabar S, Kurzweil AM. Training in neurology: identifying and addressing struggling colleagues in the era of physician burnout. Neurology. 2020;95(17):796-799. doi:10.1212/wnl.0000000000010601. 32788253

[19] Gaba DM. Crisis resource management and teamwork training in anaesthesia. Br J Anaesth. 2010;105(1):3-6. doi:10.1093/bja/aeq124. 20551023

[20] Bleetman A, Sanusi S, Dale T, Brace S. Human factors and error prevention in emergency medicine. Emerg Med J. 2012;29(5):389-393. doi:10.1136/emj.2010.107698. 21565880

[21] Rajendram P, Notario L, Reid C, et al. Crisis resource management and high-performing teams in hyperacute stroke care. Neurocrit Care. 2020;33(2):338-346. doi:10.1007/s12028-020-01057-4. 32794144 PMC7426067

[22] Ajmi SC, Advani R, Fjetland L, et al. Reducing door-to-needle times in stroke thrombolysis to 13 min through protocol revision and simulation training: a quality improvement project in a Norwegian stroke centre. BMJ Qual Saf. 2019;28(11):939-948. doi:10.1136/bmjqs-2018-009117. 31256015

[23] Dworetzky BA, Peyre S, Bubrick EJ, et al. Interprofessional simulation to improve safety in the epilepsy monitoring unit. Epilepsy Behav. 2015;45:229-233. doi:10.1016/j.yebeh.2015.01.018. 25812939

[24] Stredny CM, Abend NS, Loddenkemper T. Towards acute pediatric status epilepticus intervention teams: do we need “seizure codes”? Seizure. 2018;58:133-140. doi:10.1016/j.seizure.2018.04.011. 29702411

[25] Patel RA, Mohl L, Paetow G, Maiser S. Acute neuromuscular respiratory weakness due to acute inflammatory demyelinating polyneuropathy (AIDP): a simulation scenario for neurology providers. MedEdPORTAL. 2019;15:10811. doi:10.15766/mep_2374-8265.10811. 30931390 PMC6415011

[26] Keller JM, Steinbach TC, Adamson R, et al. ICU emergencies simulation curriculum for critical care fellows: neurologic emergencies. MedEdPORTAL. 2019;15:10813. doi:10.15766/mep_2374-8265.10813. 31139732 PMC6489375

[27] Koff A, Burns R, Auerbach M, et al. Pediatric emergency medicine didactics and simulation (PEMDAS) telesimulation series: hyperleukocytosis. MedEdPORTAL. 2021;17:11205. doi:10.15766/mep_2374-8265.11205. 34963902 PMC8666409

[28] Vora S, Li J, Kou M, et al. ACEP SimBox: a pediatric simulation-based training innovation. Ann Emerg Med. 2021;78(3):346-354. doi:10.1016/j.annemergmed.2021.03.040. 34154842

[29] London ZN, Gallagher GW, Ebright MJ. A self-study curriculum in electromyography and nerve conduction studies for residents and fellows. MedEdPORTAL. 2017;13:10581. doi:10.15766/mep_2374-8265.10581. 30800783 PMC6338186

[30] Vera M, Kattan E, Cerda T, et al. Implementation of distance-based simulation training programs for healthcare professionals: breaking barriers during COVID-19 pandemic. Simul Healthc. 2021;16(6):401-406. doi:10.1097/sih.0000000000000550. 33913677

[31] Austin A, Rudolf F, Fernandez J, et al. COVID-19 educational innovation: hybrid in-person and virtual simulation for emergency medicine trainees. AEM Educ Train. 2021;5(2):e10593. doi:10.1002/aet2.10593. 33786409 PMC7995095

[32] Valentine D, Kurzweil A, Zabar S, Lewis A. Objective structured clinical exams (OSCE) are a feasible method of teaching how to discuss a nonepileptic seizure diagnosis. Epilepsy Behav. 2019;100(A):106526. doi:10.1016/j.yebeh.2019.106526. 31654939

[33] Hayden EM, Khatri A, Kelly HR, Yager PH, Salazar GM. Mannequin-based telesimulation: increasing access to simulation-based education. Acad Emerg Med. 2018;25(2):144-147. doi:10.1111/acem.13299. 28846175

[34] Hatcher-Martin JM, Adams JL, Anderson ER, et al. Telemedicine in neurology: telemedicine work group of the American Academy of Neurology update. Neurology. 2020;94(1):30-38. doi:10.1212/wnl.0000000000008708. 31801829

[35] Afshari M, Witek NP, Galifianakis NB. Education research: an experiential outpatient teleneurology curriculum for residents. Neurology. 2019;93(4):170-175. doi:10.1212/wnl.0000000000007848. 31332085

[36] Zha AM, Chung LS, Song SS, Majersik JJ, Jagolino-Cole AL. Training in neurology: adoption of resident teleneurology training in the wake of COVID-19: telemedicine crash course. Neurology. 2020;95(9):404-407. doi:10.1212/wnl.0000000000010029. 32554768 PMC7538229

[37] Tipton PW, D'Souza CE, Greenway MRF, et al. Incorporation of telestroke into neurology residency training: “time is brain and education.” Telemed J E Health. 2020;26(8):1035-1042. doi:10.1089/tmj.2019.0184. 31821116

[38] Jagolino-Cole AL, Bozorgui S, Ankrom CM, et al. Benchmarking telestroke proficiency: page-to-needle time among neurovascular fellows and attendings. Stroke. 2017;48(9):2618-2620. doi:10.1161/strokeaha.117.017394. 28754832

[39] Bhatti D, Bertoni J. Distance learning for neurology: experiences from mini-fellowship in movement disorders. Pakistan J Neurol Sci. 2020;15(1):9.

[40] Deorari AK, Paul VK, Singh M, Vidyasagar D. Impact of education and training on neonatal resuscitation practices in 14 teaching hospitals in India. Ann Trop Paediatr. 2001;21(1):29-33. doi:10.1080/02724930123814. 11284243

[41] Niermeyer S. From the Neonatal Resuscitation Program to Helping Babies Breathe: global impact of educational programs in neonatal resuscitation. Semin Fetal Neonatal Med. 2015;20(5):300-308. doi:10.1016/j.siny.2015.06.005. 26265602

[42] Pitt MB, Eppich WJ, Shane ML, Butteris SM. Using simulation in global health: considerations for design and implementation. Simul Healthc. 2017;12(3):177-181. doi:10.1097/sih.0000000000000209. 27902533

[43] Dewey JJ, Chiota-McCollum N, Barratt D, et al. Introducing the neurology milestones 2.0. Neurology. 2022;98(9):366-372. doi:10.1212/wnl.0000000000013312. 35017311

[44] Regan L, Cope L, Omron R, Bright L, Bayram JD. Do end-of-rotation and end-of-shift assessments inform clinical competency committees' (CCC) decisions? West J Emerg Med. 2018;19(1):121-127. doi:10.5811/westjem.2017.10.35290. 29383066 PMC5785178

[45] Mikhaeil-Demo Y, Holmboe E, Gerard EE, et al. Simulation-based assessments and graduating neurology residents' milestones: status epilepticus milestones. J Grad Med Educ. 2021;13(2):223-230. doi:10.4300/JGME-D-20-00832.1. 33897956 PMC8054597

[46] Pergakis MB, Chang WTW, Tabatabai A, et al. Simulation-based assessment of graduate neurology trainees' performance managing acute ischemic stroke. Neurology. 2021;97(24):e2414-e2422. doi:10.1212/wnl.0000000000012972. 34706974

[47] Mikhaeil-Demo Y, Barsuk JH, Culler GW, et al. Use of a simulation-based mastery learning curriculum for neurology residents to improve the identification and management of status epilepticus. Epilepsy Behav. 2020;111:107247. doi:10.1016/j.yebeh.2020.107247. 32603805

[48] Morris NA, Chang WT, Tabatabai A, et al. Development of neurological emergency simulations for assessment: content evidence and response process. Neurocrit Care. 2021;35(2):389-396. doi:10.1007/s12028-020-01176-y. 33479919

[49] Korres S, Balatsouras DG, Zournas C, Economou C, Gatsonis SD, Adamopoulos G. Periodic alternating nystagmus associated with Arnold-Chiari malformation. J Laryngol Otol. 2001;115(12):1001-1004. doi:10.1258/0022215011909602. 11779333

[50] Erro R, Reich SG. Rare tremors and tremors occurring in other neurological disorders. J Neurol Sci. 2022;435:120200. doi:10.1016/j.jns.2022.120200. 35220114

[51] Cision. Gaumard Scientific to Unveil the World's Most Advanced Multidisciplinary Patient Simulator, HAL S5301. PR Newswire; 2022. Accessed February 28, 2022. prnewswire.com/news-releases/gaumard-scientific-to-unveil-the-worlds-most-advanced-multidisciplinary-patient-simulator-hal-s5301-301461445.html.

[52] Sandrone S, Carlson CE. Future of neurology & technology: virtual and augmented reality in neurology and Neuroscience Education. Neurology. 2021;97(15):740-744. doi:10.1212/wnl.0000000000012413. 34187858

[53] Chaballout B, Molloy M, Vaughn J, Brisson R III, Shaw R. Feasibility of augmented reality in clinical simulations: using Google glass with manikins. JMIR Med Educ. 2016;2(1):e2. doi:10.2196/mededu.5159. 27731862 PMC5041361

[54] Cook DA, Erwin PJ, Triola MM. Computerized virtual patients in Health Professions Education: a systematic review and meta-analysis. Acad Med. 2010;85(10):1589-1602. doi:10.1097/acm.0b013e3181edfe13. 20703150

[55] Tang KS, Cheng DL, Mi E, Greenberg PB. Augmented reality in medical education: a systematic review. Can Med Educ J. 2020;11(1):e81-e96. doi:10.36834/cmej.61705. PMC7082471 32215146

[56] Barsuk JH, Cohen ER, Feinglass J, McGaghie WC, Wayne DB. Use of simulation-based education to reduce catheter-related bloodstream infections. Arch Intern Med. 2009;169(15):1420-1423. doi:10.1001/archinternmed.2009.215. 19667306

[57] Gaubert S, Blet A, Dib F, et al. Positive effects of lumbar puncture simulation training for medical students in clinical practice. BMC Med Educ. 2021;21(1):18. doi:10.1186/s12909-020-02452-3. 33407416 PMC7789333

[58] Odom M, Gomez JR, Danelson KA, Sarwal A. Development of a homemade spinal model for simulation to teach ultrasound guidance for lumbar puncture. Neurocrit Care. 2019;31(3):550-558. doi:10.1007/s12028-019-00779-4. 31313141

[59] Lerner DJ, Gifford SE, Olafsen N, Mileto A, Soloff E. Lumbar puncture: creation and resident acceptance of a low-cost, durable, reusable fluoroscopic phantom with a fluid-filled spinal canal for training at an academic program. AJNR Am J Neuroradiol. 2020;41(3):548-550. doi:10.3174/ajnr.a6439. 32079597 PMC7077888

[ref60] eReferences are listed at links.lww.com/NE9/A20.

